# Associations between Shared Sanitation, Stunting and Diarrhoea in Low-Income, High Density Urban Neighbourhoods of Maputo, Mozambique - a Cross-Sectional Study

**DOI:** 10.1007/s10995-024-03924-4

**Published:** 2024-03-01

**Authors:** Laura Braun, Amy MacDougall, Trent Sumner, Zaida Adriano, Edna Viegas, Rassul Nalá, Joe Brown, Jackie Knee, Oliver Cumming

**Affiliations:** 1 Department of Disease Control, London School of Hygiene and Tropical Medicine, London, UK; 2 Department of Medical Statistics, London School of Hygiene and Tropical Medicine, London, UK; 3 School of Civil and Environmental Engineering, Georgia Institute of Technology, Atlanta, USA; 4 WE Consult ltd, Maputo, Mozambique; 5 Instituto Nacional de Saúde Maputo, Maputo, Mozambique; 6 Department of Environmental Sciences and Engineering, Gillings School of Public Health, University of North Carolina at Chapel Hill, Chapel Hill, NC USA

**Keywords:** Shared sanitation, Diarrhoea, Stunting, Urban

## Abstract

**Introduction:**

Shared sanitation facilities are used by over 500 million people around the world. Most research evidence indicates that shared sanitation conveys higher risk than household sanitation for many adverse health outcomes. However, studies often fail to account for variation between different types of shared facilities. As informal housing development outpaces sanitation infrastructure, it is imperative to understand which components of shared facilities may mitigate the health risks of shared sanitation use.

**Methods:**

This cross-sectional study determines whether sanitation improvement or compound hygiene were associated with stunting or diarrhoeal prevalence in children under five living in Maputo, Mozambique who rely on shared sanitation facilities. The study uses logistic and linear multivariable regression analysis to search for associations and control for potential confounding factors.

**Results:**

346 children (43.9%) in the study population were stunted. Each unit increase in sanitation score was associated with an approximate decrease of 22% in the odds of stunting (OR: 0.78, CI: 0.66, 0.92), and an increase in height of 0.23 height-for-age z-scores (CI: 0.10, 0.36). There was no evidence that the compound hygiene score was associated with height as measured by stunting (OR: 1.05, CI: 0.87, 1.26) or z-score (-0.06, CI: -0.21, 0.09). Neither sanitation nor compound hygiene score were associated with diarrhoea in the population.

**Conclusions:**

Use of an improved shared latrine is associated with decreased odds of stunting. There is no evidence of an association between latrine improvement and diarrhoea. Further investigation is necessary to isolate attributes of shared sanitation facilities that may reduce health risks.

**Supplementary Information:**

The online version contains supplementary material available at 10.1007/s10995-024-03924-4.

## Significance

As urban growth increases and expansion of informal housing outpaces sanitation infrastructure, it is imperative to understand which components of a shared facility may mitigate the health risks associated with shared sanitation use. Thereby, an evidence base can be built so that recommendations can be adopted by the WHO.

**Supplementary Information:**

The online version contains supplementary material available at 10.1007/s10995-024-03924-4.

## Introduction

In 2019, the World Health Organization (WHO) estimated that 105 million disability-adjusted life-years (DALYs) were attributable to poor water, sanitation, and hygiene (WASH) conditions (Prüss-Ustün et al., 2019). An estimated 829,000 diarrhoeal deaths in low- and middle- income countries (LMICs) were attributed to unimproved WASH conditions, with 432,000 of these deaths linked to poor sanitation – unsafe management of human excreta – alone (Prüss-Ustün et al., 2019). Unsafe sanitation enables the transmission of faecal-oral pathogens and is a risk factor for numerous adverse health outcomes, including gastrointestinal diseases (Schorling et al., 1990), undernutrition (Buttenheim, 2008), and stunting (Assis et al., 2005; Cumming and Cairncross, 2016).

Stunting – being too short for one’s age – is the most prevalent form of undernutrition and affects approximately 149 million children under the age of five globally (World Health Organization, United Nations Children’s Fund, World Bank, 2021). It is associated with poor cognitive development (Caulfield et al., 2006) and increased morbidity and mortality (Black et al., 2008; Olofin et al., 2013; Pelletier et al., 1995). Sanitation conditions are thought to be an important driver of stunting, alongside inadequate nutritional intake and other chronic infections not caused by poor sanitation (Prendergast and Humphrey, 2014). Most childhood stunting is believed to be preventable, making early childhood a critical window for implementing interventions (Stewart et al., 2013). Repeated bouts of diarrhoea are thought to be a key mediating pathway between sanitation and stunting, and pooled analyses have shown that children with more past episodes of diarrhoea have increased odds of stunting at 24 months of age (Checkley et al., 2008). Subclinical enteric infections may also hinder growth and development outcomes, affecting the gut’s absorptive capacity and limiting the effects of nutritional and medical interventions (Prendergast and Humphrey, 2014; Rogawski et al., 2018). If both clinical and subclinical enteric infections cause poor nutrient absorption and thereby stunted linear growth, then access to improved sanitation facilities might plausibly improve growth outcomes by reducing exposure to enteric pathogens.

The WHO/UNICEF Joint Monitoring Programme for Water Supply and Sanitation (JMP), which monitors progress on Sustainable Development Goal (SDG) WASH targets, uses a ‘ladder’ approach to categorise levels of sanitations service from “open defecation”, to “limited”, to “basic” and to “safely-managed services” (World Health Organization & United Nations Children’s Fund, 2021). For sanitation to be considered as a basic service, it must be “improved” – meaning that they are designed to hygienically separate excreta from human contact - and cannot be shared by more than one household. Where a basic sanitation facility is shared by more than one household it is considered “limited” as it is assumed based on some evidence that shared facilities carry a higher risk with regard to infectious disease compared to private household (Fuller et al., 2014; Heijnen et al., 2014; Shultz et al., 2009; Sobel et al., 2004). However, one recent multi-country study found that the sharing of sanitation facilities by multiple households was only associated with increased risk of diarrhoea in some settings (Baker et al., 2016) Shared sanitation facilities are used by over 580 million people around the world (World Health Organization & United Nations Children’s Fund, 2021) and this number has been growing among the urban population in many countries due in part to the limited space available for construction as well as the cost (World Health Organization, & United Nations Children’s Fund, 2015). As urban growth increases and expansion of informal housing outpaces sanitation infrastructure, it is imperative to understand which components of a shared facility may mitigate the health risks associated with shared sanitation use. Thereby, an evidence base can be built so that recommendations can be adopted by the WHO.

The aim of this study was to determine which sanitation-related factors were most strongly associated with increased prevalence of stunting and diarrheal disease among young children using shared sanitation facilities living in urban environments of Maputo, Mozambique. An approach which combined individual components of shared sanitation into a cumulative score was contrasted with one which considered each component as a separate risk factor.

## Methods

### Study and Setting

This study uses baseline data from a cross-sectional survey conducted as part of the Maputo Sanitation (MapSan) Trial, a controlled, before-and-after trial of a sanitation intervention in urban Maputo, Mozambique (ClinicalTrials.gov Identifier NCT02362932) (Brown et al., 2015).

The study took place in neighbourhoods of Maputo where only approximately a quarter of human waste is safely managed (Blackett et al., 2014). All study participants lived in compounds that primarily used shared sanitation between households according to the inclusion criteria of the MapSan trial (Brown et al., 2015). Sanitation systems mostly included latrines with different attributes, such as vent pipes and slabs. At baseline, the study population comprised children aged 29 days to 48 months living in a compound enrolled in the MapSan trial. As described previously (Knee et al., 2021), only compounds meeting the following inclusion criteria were included: (1) compounds in urban neighbourhoods of Maputo; (2) residents using shared sanitation in poor condition as determined by a project sanitation engineer; (3) sanitation is shared with a minimum of 12 people from at least two different households; (4) located near a legal piped water connection; (5) residents demonstrate an interest in improved sanitation. Intervention compounds also had to (6) be willing to contribute financially to construction costs, (7) have access to a legal piped water supply, (8) be accessible for transportation of construction materials and tank-emptying activities, and (9) have groundwater level deep enough for construction of a septic tank. Agreement was sought from the heads (“chefes”) of eligible compounds to be included in the study and, if they agreed, the compounds were included and subsequently visited by the research team.

### Data Collection

Data were collected between February 2015 and February 2016. Data for each compound were collected over two days by field researchers through observations and two questionnaires (one household and one child health questionnaire). The guardian or parent of the participating child provided written informed consent prior to any individual data being collected. Field researchers were blind to the current study hypotheses. ​Data were collected on phones using the Magpi mobile data collection tool (“DataDyne,”; 2017).

Household and compound sanitation variables that could be visually observed were recorded by a fieldworker using structured observations to limit observer bias. All other variables were obtained via questionnaires. Questions pertaining to the household were answered by the household head in most cases, and questions pertaining to the child’s health were answered by the child’s primary caregiver. Child height was obtained by direct measurement by field enumerators trained in standard methods (World Health Organization, 2008b). Child diarrhoea was reported by the caretaker with a 1-week recall. As often as possible, household and child questionnaires took place on the same day as anthropometric data collection. Sometimes children were not available at the time of the child questionnaire, in which case enumerators returned the following day to complete questionnaires. Each questionnaire was answered by one person only. In rare cases, there was a longer period between completing household and child surveys, up to a period of weeks.

### Measures

The primary exposures of interest were: (1) sanitation score, i.e., the presence of specific physical components of the latrine’s infrastructure that plausibly reduce the risk of transmission of pathogens via faecal-oral routes, and (2) compound hygiene score, i.e., the absence of visible faeces on the compound and surrounding the latrine. We hypothesised that both scores would be associated with increased probability of stunting and diarrhoea. Both scores comprised multiple individual components frequently used in sanitation trials, as listed below.

Sanitation score was defined as the sum of four binary variables: presence of a covering for the latrine drop-hole (yes/no), presence of a covered ventilation pipe on the drop-hole (yes/no), presence of pedestal masonry or a tile slab on the latrine drop-hole (yes/no), and presence of stone walls surrounding the latrine (yes/no). These design features help avoid direct and passive contact with excreta (World Health Organization, 2018). Specifically, the presence of a slab for a pit latrine differentiates unimproved from improved sanitation facilities according to the WHO/UNICEF JMP sanitation ladder (World Health Organization & United Nations Children’s Fund, 2021). Drop-hole covers prevent rodents or insects from entering, and together with a covered ventilation pipe help reduce flies and odours. The stone wall superstructures provides privacy and helps prevent rainwater or animals from entering (World Health Organization, 2018). In this construction, “Yes” responses were coded 1 while “No” responses were coded 0, resulting in a score of 4 for the most improved latrines and 0 for unimproved latrines.

Compound hygiene comprised three binary variables: visible faeces (human or animal) or soiled diapers on the compound grounds (yes/no), visible presence of wastewater leaking from the latrine (yes/no), and susceptibility to flooding in the rainy season (yes/no; obtained via interview). These measures have been recorded in similar studies (Arnold et al., 2013; Pickering et al., 2015) as potential confounding variables for the effect of a sanitation intervention on diarrhoea or stunting (Baker et al., 2014; Baker, O’Reilly, Baker et al., 2016a, b). Each component contributed 1 point to the compound hygiene score, for a final possible score ranging from 0 to 3. “No” responses were coded 1, while “Yes” responses were coded 0, so that a score of 3 corresponded to the most hygienic compounds while a score of 0 corresponded to the least hygienic compounds. Similar additive methods of index construction have been used previously in peer-reviewed sanitation studies (Jenkins et al., 2014; Schreiner, 2013).

Child height was obtained by direct measurement at enrolment and entered in Magpi. Height and age were recorded by the field researcher with the child wearing minimal clothing and no shoes as per standard protocols (World Health Organization, 2008a). Height-for-age z-scores were calculated according to the WHO child growth standards (WHO Multicentre Growth Reference Study Group and Mercedes de Onis, 2006). For children under two years of age, recumbent length using a length board was recorded instead of standing height as per WHO protocol. Stunting was defined as having a height-for-age z-score below − 2 (Black et al., 2008; Marriott et al., 2012). Diarrhoeal outcome measures were obtained by interviewing the child’s mother (or primary caregiver in cases when the mother was not available) about diarrhoeal prevalence in the previous seven days, defined as at least three loose stools per day.

Wealth was defined using a validated Simple Poverty Scorecard constructed for Mozambique (Schreiner, 2013) based on household assets, construction material and energy source, which calculates the likelihood of being below the poverty line for each household. Relative socioeconomic status (SES) used in the regression models was established by dividing the poverty score into tertiles. Household crowding was obtained by calculating the number of individuals per bedroom in the household. The crowding variable was a binary indicator of whether the number of individuals per bedroom was greater than three. There was no upper cut-off.

### Statistical Analysis

Height-for-age z-scores were plotted and examined for outliers. Extreme z-scores of <-6 or > 6 were censored, according to WHO standards for children 0–60 months (World Health Organization and United Nations Children’s Fund, 2019). The outcomes, height-for-age z-score, stunting and self-reported diarrhoea, were analysed in separate multivariable models. For the main analysis, each model was run with the composite scores for compound hygiene and sanitation as well as possible confounders, determined a priori. In a secondary analysis, the components of the composite variables were entered individually into two models, one for compound sanitation and the other for compound hygiene.

For height-for-age z-scores, a mixed effects linear regression was used. For the binary outcomes, stunting and self-reported diarrhoea, a mixed effects logistic regression was used. In all models, a random effect at the level of the compound was included to account for within compound correlation.

The models for height and stunting included the following covariates: age, sex, wealth tercile, indicator of whether primary carer had completed primary school, binary indicator of any breastfeeding, binary indicator of whether a tap for water was present in the compound. A natural cubic spline with two knots was used for age. The low number of diarrhoeal events limited the number of parameters available for use in the regression model; age, sex, and wealth tercile were selected.

A complete case analysis was conducted, assuming that all missing values were missing at random. All analyses were carried out using the R statistical software (R Core Team, 2019) using the ‘lme4’ package (Bates et al., 2014).

## Results

### Population Description

A total of 987 children were eligible for this analysis of which 169 (17%) were excluded for having no height measurement. A further 29 were missing either a complete compound hygiene or sanitation score (4%) as at least one of the seven binary questions was left unanswered. After these exclusions, a total of 789 children from 425 compounds were included. Most children were under the age of two (54%), with only 1.6% being four years old. Most caretakers reported having completed primary school (54.1%) and having at least one water point (77.4%). A third of children were breastfed at the time of the study (Table 1).

The median height-for-age z-score was − 1.7 (IQR: -2.9, -0.7, Table 1) and almost half of children were classified as being stunted (43.9%, *n* = 346). Approximately one in seven (14.3%, *n* = 133) children were reported as having experienced diarrhoea in the previous week.

The absence of wastewater near latrines and flooding of compounds in the rainy season were reported in approximately one third of children’s compounds (35.0%, 36.2% respectively, Table 1). Faeces or soiled diapers were not visible in around half of children’s compounds (51.2%). The most common improvement was a drop-hole covering, present in half of latrines (53.1% with drop-hole covered). Around one third were recorded to have a pedestal masonry, or walls made from stone (36.8% pedestal masonry in latrine; 30.5% latrine walls made of stone). A small proportion (13.8%) were recorded as having pit ventilation, which helps reduce odour and flies.

**Table 1:** Population description for children with complete data

*Total n*	
n	789
Number of compounds	425
*Demographics*	
Age, years (%)	
0	203 (25.7)
1	223 (28.3)
2	210 (26.6)
3	140 (17.7)
4	13 (1.6)
Sex (n, % female)	400 (50.7)
Poverty score, median [IQR]	43.0 [36.0, 50.0]
Carer finished primary school (%)	427 (54.1)
Crowded (%)	144 (18.3)
Child currently breastfed (exclusive or complementary) (%)	251 (31.8)
At least one water point on compound (%)	611 (77.4)
*Outcomes*	
Height-for-age z-score (median [IQR])	-1.7 [-2.9, -0.7]
Stunted (%)	346 (43.9)
Carer-reported child diarrhoea within the last week (%)	113 (14.3)
*Compound hygiene*	
No wastewater near latrines or leaking from latrines (%)	276 (35.0)
No faeces visible on compound (human/animal/soiled diaper) (%)	404 (51.2)
No compound floods in the rainy season (%)	286 (36.2)
*Compound sanitation*	
Covered drop-hole (%)	419 (53.1)
Ventilation piped on latrine (%)	109 (13.8)
Pedestal masonry in latrine (%)	290 (36.8)
Latrine walls made of stone (%)	241 (30.5)

**Fig. 1:**
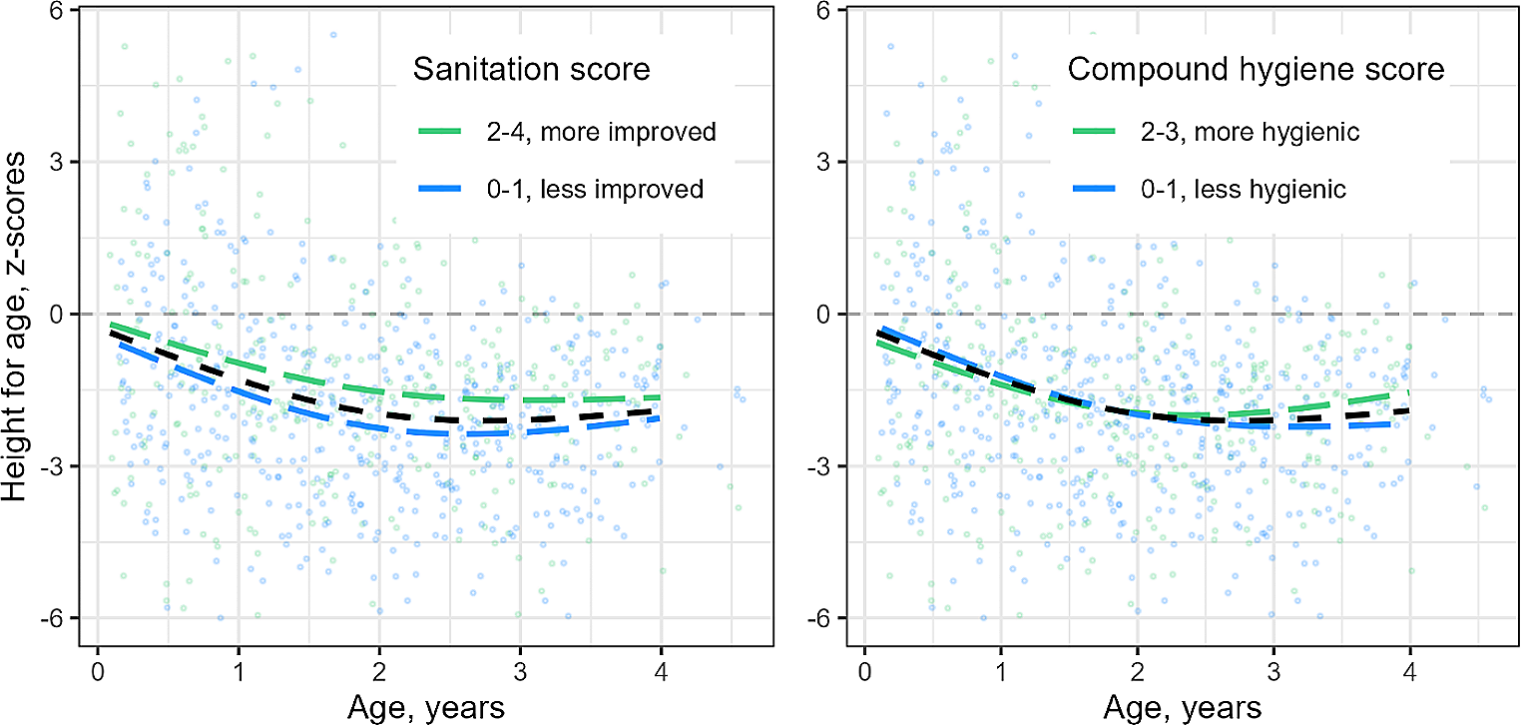
**a** and **b** Height-for-age z-scores by age with a smoothed average curve by sanitation score (1**a**) and compound hygiene score (1**b**). The dashed lines indicate that this is cross-sectional, rather than longitudinal data, so the average line does not represent a growth curve which an individual might follow over time. The overall average is shown in black

The height-for-age z-scores decreased on average with age (Fig. 1, black dashed lines) and were highest in under ones, though with a wide degree of variation. After a decline to age three years height-for-age scores remained stable to age four. In those with a sanitation score of at least 2, the average height-for-age z-score did not decrease as much as for those with a score of 0 or 1 (Fig. 1a). Median height-for-age z-score did not increase substantially with higher compound hygiene scores (-1.8 median z-score for compound hygiene score of 0; -1.7 for a score of 3 (Table 2); dashed lines not substantially different, Fig. 1b). The proportion with carer reported diarrhoea did decrease, from 18.9 to 12.6% for scores 3 and 0, respectively (Table 2).

More than half the children lived in compounds with highly unimproved sanitary conditions (sanitation score ≤1) (Table 2). Approximately the same number had a hygiene score of 1 or lower. The proportion of children classed as stunted decreased as sanitation score increased (52.4% stunted with improvement score of 0, 38.4% score of 4, Table 2). Median height-for-age z-score increased from − 2.1 for those with a score of zero to -1.6 with a score of 4. The proportion with carer-reported diarrhoea by sanitation improvement did not display a clear trend.

**Table 2:** Outcomes by composite scores

	n (%)	Stunted, n (%)	Height-for-age z-score, median [IQR]	Diarrhoea, n (%)
*Sanitation score* *Higher = more improved*				
0	252 (31.9)	132 (52.4)	-2.1 [-3.4, -1.1]	33 (13.1)
1	219 (27.8)	100 (45.7)	-1.7 [-2.9, -0.7]	34 (15.5)
2	187 (23.7)	65 (34.8)	-1.5 [-2.5, -0.3]	28 (15.0)
3	58 (7.4)	21 (36.2)	-1.3 [-2.8, 0.1]	3 (5.2)
4	73 (9.2)	28 (38.4)	-1.6 [-2.4, -0.4]	15 (20.5)
*Compound hygiene score* *Higher = more hygienic*				
0	249 (31.6)	110 (44.2)	-1.8 [-2.8, -0.7]	47 (18.9)
1	225 (28.5)	100 (44.4)	-1.7 [-3.1, -0.8]	29 (12.9)
2	204 (25.8)	93 (45.6)	-1.8 [-3.0, -0.7]	23 (11.3)
3	111 (14.1)	43 (38.7)	-1.7 [-2.6, -0.6]	14 (12.6)

### Analysis

There was evidence from the analysis that the sanitation score was associated with height. Each one-point increase in sanitation score was associated with an approximate decrease of 22% in the odds of stunting (OR: 0.78, CI: 0.66, 0.92, Table 3), and an increase in height of 0.23 height-for-age z-scores (CI: 0.10, 0.36). There was no evidence that the compound hygiene score was associated with height as measured by stunting (OR: 1.05, CI: 0.87, 1.26) or z-score (-0.06, CI: -0.21, 0.09).

In the models including the individual components of each score, it was clear that the association between sanitation score and height was driven by drop-hole covering. The odds of stunting were approximately halved for those with a drop-hole covering (OR: 0.49, CI:0.32, 0.75), and height increased by 0.62 height-for-age z-scores (CI: 0.30, 0.94, Table 3).

**Table 3:** Results of regressions of stunting and height-for-age z-score on composite compound hygiene, sanitation scores and confounders; and individual components of these scores. Logistic regressions were used for stunting, and linear regressions for height-for-age z-score. All models were adjusted for covariates described in the methods section and full results are in the supplement

	Stunting		Height-for-age Z-score	
	OR (CI)	p-value	Coefficient (CI)	p-value
**Models I: composite scores**				
Compound hygiene score	1.05 (0.87, 1.26)	0.63	-0.06 (-0.21, 0.09)	0.41
Sanitation score	0.78 (0.66, 0.92)	< 0.01	0.23 (0.10, 0.36)	< 0.01
**Models II: individual components**				
*Compound hygiene score*				
No wastewater near latrines or leaking from latrines	1.16 (0.74, 1.82)	0.52	-0.02 (-0.37, 0.33)	0.89
No faeces visible on compound	0.96 (0.61, 1.51)	0.85	-0.11 (-0.46, 0.24)	0.54
No compound floods in the rainy season	1.07 (0.72, 1.59)	0.74	-0.08 (-0.39, 0.23)	0.62
*Sanitation score*				
Drop-hole covering	0.49 (0.32, 0.75)	< 0.01	0.62 (0.30, 0.94)	< 0.01
Ventilation piped on latrine	0.92 (0.48, 1.76)	0.81	0.04 (-0.45, 0.54)	0.86
Pedestal masonry in latrine	0.86 (0.56, 1.32)	0.49	0.19 (-0.14, 0.51)	0.26
Latrine walls made of stone	1.07 (0.66, 1.72)	0.79	0.01 (-0.36, 0.38)	0.97

In contrast, compound hygiene score was associated with carer-reported diarrhoea, while sanitation score was not. The odds of diarrhoea decreased by approximately 35% for each increase in the compound hygiene score (OR: 0.74, CI: 0.57, 0.97, Table 4). The odds ratio for association between sanitation and diarrhoea score was 1.12 (CI: 0.90, 1.39).

Similarly, the association between compound hygiene and diarrhoea was driven by a single measure: faeces visible on compound. This included human or animal faeces, or soiled diapers. The odds of diarrhoea were more than halved when no faeces were recorded (OR: 0.35, CI: 0.18, 0.69). There was no evidence that the other components of either score were associated with diarrhoea.

**Table 4:** Results of regressions of carer-reported diarrhoea on composite compound hygiene and sanitation scores, and individual components of these scores. Mixed effects logistic regressions were used. All models were adjusted for covariates described in the methods section and full results are in the supplement

Diarrhoea		
	OR (CI)	p-value
**Models I: composite scores**		
*Compound hygiene score*	0.74 (0.57, 0.97)	0.03
*Sanitation score*	1.12 (0.90, 1.39)	0.32
**Models II: individual components**		
*Compound hygiene score*		
No wastewater near latrines or leaking from latrines	1.16 (0.61, 2.21)	0.66
No faeces visible on compound	0.35 (0.18, 0.69)	< 0.01
No compound floods in the rainy season	1.04 (0.60, 1.81)	0.89
*Sanitation score*		
Drop-hole covering	1.47 (0.83, 2.60)	0.19
Ventilation piped on latrine	1.26 (0.52, 3.06)	0.61
Pedestal masonry in latrine	1.28 (0.71, 2.31)	0.40
Latrine walls made of stone	0.82 (0.42, 1.61)	0.56

## Discussion

In a population living in a low-income urban environment, improved latrines were found to be associated with greater height-for-age in children under four years of age. This association was found to be driven primarily by the presence of a drop-hole cover, which approximately halved the odds of stunting and increased height-for-age z-score. Drop-hole covers help reduce flies and odour, and thereby may also make latrines more user-friendly. Flies have been identified as potentially important mechanical vectors for the transmission of enteric pathogens in urban Maputo (Capone et al., 2023). In addition, the MapSan trial previously reported that drop-hole covering was associated with reduced risk of bacterial and protozoan infection, which may explain the link to stunting (Knee et al., 2018). Most of these infections were asymptomatic, and research indicates that non-diarrhoeal asymptomatic carriage is associated with stunting (Rogawski et al., 2018). The wider environment, as measured by the compound hygiene score, was not found to be associated with height. This score is likely to be more variable and stochastic than a structural measurement such as latrine improvement and may hence not be a good predictor of stunting prevalence.

These findings were reversed for carer-reported diarrhoea. Sanitation score was not found to be associated with carer-reported diarrhoea, while compound hygiene score was. In particular, the absence of visible faeces or soiled diapers on the compound was associated with a decreased odd of diarrhoea. This highlights the importance of safe use of sanitation facilities, as the presence of improved latrines alone does not prevent faecal contamination of the environment.

Composite scores were used to assess the cumulative effect of latrine and compound hygiene. We hypothesised that the cumulative association of the selected components may have been greater than the sum of the component odds. However, we did not find this to be the case for either of the scores used in this study. Rather than each measure of compound hygiene or sanitation having a small association with outcome, which contributed to a greater association with outcome when combined, individual components were either independently associated strongly with outcome or not at all. The sanitation and hygiene scores are based on a small number of important variables, which differs to indexes such as the Localized Sanitation Status Index (LSSI) which is calculated from 20 variables (Capone et al., 2019). An unweighted index with few important variables may be useful in settings where full sanitary surveys are unlikely to be completed.

Overall, the results show that improved latrines were associated with stunting but not diarrhoea. This finding differs from several studies which have found an association between sanitation and diarrhoea (Baker et al., 2016; Gunther and Fink, 2010; Prüss-Ustün et al., 2019; Prüss-Ustün et al., 2014), and those which have found no effect of sanitation on childhood diarrhoea (Humphrey et al., 2019; Luby et al., 2018; Null et al., 2018). Nonetheless, a recent randomised trial found a strong effect of a sanitation intervention on stunting but not on diarrhoea, though this was in a rural setting and used the community-led total sanitation approach (Pickering et al., 2015). These findings may be driven by the high prevalence of subclinical, non-diarrhoeal infections in children living in the study area, as found in this dataset (Knee et al., 2018). This asymptomatic carriage of enteric infections such as *Giardia* and *Shigella* has been associated with stunting (Rogawski et al., 2018). Although not fully defined, the mechanism that links subclinical enteric infection and stunting likely involves environmental enteric dysfunction (George et al., 2018) and intestinal inflammation (Amour et al., 2016), permeability and malabsorption (Rogawski et al., 2017).

### Limitations

This study did not collect information on either child or caregiver hand hygiene practices, which are associated with both diarrhoea (Alam et al., 1989; Clasen et al., 2007) and stunting (Schmidt, 2014). Hand hygiene practices were omitted due to issues with reporting bias, and sufficient water quantity for hygiene was deemed more objective. If hand hygiene is associated with latrine quality, the independent effect of latrine quality may be confounded. The questionnaires used in this study also did not obtain specific information on individual or household-level food availability, access, or diversity, and therefore could not control for the specific effects of diet on stunting.

Enumerators observed sanitation infrastructure to answer questions on latrine design features. However, there may be gaps between infrastructure and use, as previously found on the MapSan trial (Bick et al., 2021). Observations of behaviours and latrine operation would provide valuable information on use and access, which are critical to maximise the benefits of sanitation.

This analysis attempts to control for dietary quality using wealth as a proxy measure, hypothesising that increased wealth leads to increased food availability, access, and diversity. However, this metric is imprecise at best. Overall, there remains a risk of residual confounding as additional confounding factors were not considered, such as the mothers’ nutritional status which can impact stunting (Bhutta et al., 2008; Black et al., 2008, 2013), or food hygiene, which may mitigate pathogens present in the environment from reaching new hosts (Rogawski et al., 2017).

Caregiver-reported diarrhoea is a subjective metric and can be subject to reporter bias and misclassification of outcome (Clasen et al., 2007; Schmidt et al., 2011; Talley et al., 1994). Non-differential misclassification would bias effect estimates toward the null, which may explain the low levels of significant associations with diarrhoeal prevalence found in the study population.

This study used a cross-sectional design, so that growth patterns within individuals could not be described and causality cannot be established. Longitudinal studies and controlled trials are necessary to determine whether latrine improvement has a true effect on odds of stunting.

## Conclusion

In this cross-sectional study of children living in an environment with shared sanitation facilities, we found that improved latrines were found to be associated with greater height and reduced stunting, and greater compound hygiene score was associated with reduced odds of caregiver reported diarrhoea. This suggests that different types of shared sanitation may have important public health relevance, and variation among shared sanitation facilities is worthy of further investigation, especially through interventional study designs that limit the exposure to excreta.

### Electronic Supplementary Material

Below is the link to the electronic supplementary material.

Supplementary Material 1

## Data Availability

All data are provided in the supplementary information.
