# Supplementary material for: Associations between Shared Sanitation, Stunting and Diarrhoea in Low-Income, High Density Urban Neighbourhoods of Maputo, Mozambique - a Cross-Sectional Study
Source: Matern Child Health J. 2024 Mar 1;28(4):775–84. doi: 10.1007/s10995-024-03924-4 (PMC10963538; doi:10.1007/s10995-024-03924-4)
Supplement: Supplementary file 1 — Supplementary Material 1 [file 10995_2024_3924_MOESM1_ESM.docx]

Supplement 1: Full regression tables.

Supplemental table 1: Full results including all covariates for results of regressions of stunting and height-for-age z-score on composite compound hygiene, sanitation scores and confounders; and individual components of these scores. Logistic regressions were used for stunting, linear for height z-score. Corresponds to Table 3.

| **MODELS I: COMPOSITE SCORES** | | | | |
| --- | --- | --- | --- | --- |
|  | **STUNTING** | | **HEIGHT Z-SCORE** | |
|  | **OR (CI)** | **p-value** | **Coefficient (CI)** | **p-value** |
| **Compound hygiene score** | 1.05 (0.87, 1.26) | 0.63 | -0.06 (-0.21, 0.09) | 0.41 |
| **Sanitation score** | 0.78 (0.66, 0.92) | <0.01 | 0.23 (0.10, 0.36) | <0.01 |
| Age, years | 1.14 (0.92, 1.42) | 0.23 | (*) |  |
| Sex, male | 1.07 (0.76, 1.49) | 0.71 | 0.01 (-0.25, 0.26) | 0.96 |
| Wealth tercile | 0.81 (0.64, 1.02) | 0.07 | 0.29 (0.11, 0.46) | <0.01 |
| Carer finished primary school | 1.52 (1.06, 2.18) | 0.02 | -0.39 (-0.66, -0.12) | <0.01 |
| Child currently breastfed | 0.93 (0.55, 1.58) | 0.8 | -0.50 (-0.95, -0.04) | 0.03 |
| Crowded | 0.86 (0.52, 1.41) | 0.56 | -0.13 (-0.51, 0.25) | 0.49 |
| At least one water point on compound | 0.66 (0.43, 1.02) | 0.06 | 0.45 (0.12, 0.79) | 0.01 |
| **MODELS II: INDIVIDUAL COMPONENTS (**)** | | | | |
|  | **STUNTING** | | **HEIGHT Z-SCORE** | |
| Age, years | 1.12 (0.90, 1.39) | 0.31 | (*) |  |
| Sex, male | 1.07 (0.77, 1.50) | 0.68 | 0.00 (-0.25, 0.26) | 0.99 |
| Wealth tercile | 0.80 (0.63, 1.01) | 0.06 | 0.29 (0.12, 0.47) | <0.01 |
| Carer finished primary school | 1.54 (1.08, 2.20) | 0.02 | -0.40 (-0.67, -0.13) | <0.01 |
| Child currently breastfed | 0.90 (0.54, 1.52) | 0.71 | -0.49 (-0.95, -0.03) | 0.04 |
| Crowded | 0.85 (0.52, 1.39) | 0.51 | -0.12 (-0.50, 0.27) | 0.55 |
| At least one water point on compound | 0.67 (0.43, 1.03) | 0.07 | 0.44 (0.11, 0.78) | 0.01 |
| **Compound hygiene score** |  |  |  |  |
| No wastewater near latrines or leaking from latrines | 0.86 (0.55, 1.35) | 0.52 | 0.02 (-0.33, 0.37) | 0.89 |
| No faeces visible on compound | 1.04 (0.66, 1.64) | 0.85 | 0.11 (-0.24, 0.46) | 0.54 |
| No compound floods in the rainy season | 0.94 (0.63, 1.39) | 0.74 | 0.08 (-0.23, 0.39) | 0.62 |
| **Sanitation score** |  |  |  |  |
| Drophole covered | 0.49 (0.32, 0.75) | <0.01 | 0.62 (0.30, 0.94) | <0.01 |
| Ventilation piped on latrine | 0.92 (0.48, 1.76) | 0.81 | 0.04 (-0.45, 0.54) | 0.86 |
| Pedestal masonry in latrine | 0.86 (0.56, 1.32) | 0.49 | 0.19 (-0.14, 0.51) | 0.26 |
| Latrine walls made of stone | 1.07 (0.66, 1.72) | 0.79 | 0.01 (-0.36, 0.38) | 0.97 |
| (*) A spline was fitted for age, the coefficients for which are not readily interpretable. They are included in the appendix.  (**) These models have been adjusted for the same covariates as in part (I), the results are not shown for brevity. | | | | |

Table 4: Results of regressions of carer-reported diarrhoea on composite compound hygiene and sanitation scores and confounders; and individual components of these scores. Mixed effects logistic regressions were used.

| **DIARRHOEA** | | |
| --- | --- | --- |
| **MODELS I: COMPOSITE SCORES** | | |
|  | **OR (CI)** | **p-value** |
| **Compound hygiene score** | 0.74 (0.57, 0.97) | 0.03 |
| **Sanitation score** | 1.12 (0.90, 1.39) | 0.32 |
| Age, years | 0.72 (0.57, 0.90) | <0.01 |
| Sex, male | 0.65 (0.41, 1.03) | 0.07 |
| Wealth tercile | 0.97 (0.72, 1.30) | 0.83 |
| **MODELS II: INDIVIDUAL COMPONENTS (**)** | | |
| Age, years | 0.71 (0.56, 0.89) | <0.01 |
| Sex, male | 0.66 (0.42, 1.06) | 0.08 |
| Wealth tercile | 0.98 (0.73, 1.31) | 0.87 |
| **Compound hygiene score** |  |  |
| No wastewater near latrines or leaking from latrines | 1.16 (0.61, 2.21) | 0.66 |
| No faeces visible on compound | 0.35 (0.18, 0.69) | <0.01 |
| No compound floods in the rainy season | 1.04 (0.60, 1.81) | 0.89 |
| **Sanitation score** |  |  |
| Drophole covered | 1.47 (0.83, 2.60) | 0.19 |
| Ventilation piped on latrine | 1.26 (0.52, 3.06) | 0.61 |
| Pedestal masonry in latrine | 1.28 (0.71, 2.31) | 0.40 |
| Latrine walls made of stone | 0.82 (0.42, 1.61) | 0.56 |
